# Evaluating the psychometric properties of the Arabic version of the Groningen Frailty Indicator among Lebanese elderly people

**DOI:** 10.1186/s42506-019-0028-3

**Published:** 2019-12-23

**Authors:** Rania Khamis, Hala Sabbah, Sanaa Sabbah, Lilian Peters, Nabil Droubi, Ibtissam Sabbah

**Affiliations:** 1 Institute of Social Science, Lebanese University, Saida, Lebanon; 2 Faculty of Economic Sciences and Business Administration, Lebanese University, Nabatieh, Lebanon; 3 Doctoral School of Literature, Humanities & Social Sciences, Lebanese University, Beirut, Lebanon; 4 VU University Medical Center Amsterdam, Department of Midwifery Science AVAG, Amsterdam Public Health Research Institute, Amsterdam, The Netherlands; 5 Faculty of Public Health, Lebanese University, Saida, Lebanon

**Keywords:** Groningen Frailty Indicator, Frailty, Elderly persons, Rural and urban area, Lebanon

## Abstract

**Background:**

The levels of frailty are anticipated to increase as a result of population aging. A valid instrument is required to detect individuals at high risk of frailty. The present research aimed to assess feasibility, reliability, and construct validity of the Arabic version of Groningen Frailty Indicator (GFI) in urban and rural populations in South Lebanon.

**Methods:**

During 2015, a cross-sectional study, which enrolled 390 community-dwelling individuals aged 65 years and above, was conducted in urban and rural areas in Nabatieh in South Lebanon. The survey included questions on sociodemographic and health-related characteristics, GFI, and Vulnerable Elders Survey-13 (VES-13). The translation and cultural adaptation of the GFI followed a standardized protocol. After that, the psychometric properties of the scale (i.e., feasibility, reliability, and validity) were evaluated.

**Results:**

A total of 390 elderly filled out the questionnaire, of whom 51% were women and 70% lived in rural areas. 81.3% of elderly were identified as frail. The internal consistency of the GFI scale was high for all subscales (Cronbach’s alpha > 0.70), except the social scale (0.56). The GFI yielded statistically significant scores for subgroup analysis (known-groups validity) as higher levels of frailty were seen in older people, women, those with morbidities, and those reported poor financial status. The construct validity of the scale was supported by the significant correlation with the VES-13 (*r* = 0.73; *p* = 0.001), quality of life (*r* = − 0.22; *p* = 0.001), and self-reported health status (*r* = − 0.66; *p* = 0.001).

**Conclusion:**

This study supports the feasibility, reliability, and validity of the GFI Arabic version as a screening tool for frailty among community-dwelling elderly in South Lebanon.

## Introduction

Worldwide, the reduction in fertility rate has led to a slow pace in the population growth and higher longevity. The number of persons aged 60 or above is expected to be more than double by 2050 and to be more than triple by 2100 [1]. Population aging has led to considerable interests in issues of elderly persons [1, 2]. The factors that reduce vulnerability and disability not only have effect on the personal level of the elderly but also affect their family, society, and environment [2]. According to the World Health Organization (WHO) report, most elderly persons suffer from disability, and they are considered to have an economic and social burden on society. Undoubtedly, such issue leads to discrimination and prejudice against old persons and negatively affects policy-making and older persons’ access to health [3].

In Lebanon, the proportion of old people aged 65 years and higher did not exceed 7% in 1995 and 8% in 2015 [1]. Projections made for the year 2025 predict levels at 10% [1, 4, 5]. The assessment of the proportion of old people in each governorate has showed up wide variations: Beirut and Nabatieh have the highest proportion of elderly people and the largest old-age dependency ratio [5]. The elderly population is projected to have a profound effect on societies in Lebanon.

To identify those old people, who are almost at risk of future social and health problems, the concept of frailty has been introduced. Frailty is the loss of resources in several domains leading to the inability to respond to physical or psychological stress [6], resulting in increased risk of adverse health outcomes [7]. The levels of frailty and disability are anticipated to increase around the world [2], which poses a major burden on social and medical resources [7].

Frailty is often misconstrued to be a part of the normal aging process; the worst is that old patients are treated on the basis of their medical conditions alone, rather than accounting for their frailty status [8]. By identifying adults living with frailty, health and social care providers can implement programs focused on early detection, prevention, and management of the frailty in order to reduce future institutionalization, improve health outcomes, and enhance the quality of life of old people [9, 10].

Frailty increases with age, and women are more likely to be frail [11]. Thus, the geriatric concept of frailty is of particular interest and should be measured by a valid instrument. The Groningen Frailty Indicator (GFI) assesses the interacting losses in physical, cognitive, social, and psychological domains of functions, which leads to downward spiraling and declining reserve capacity for dealing with stressors [7, 12]. The GFI has been evaluated with good psychometric properties in Western Europe [12].

Few studies investigated the prevalence of frailty in Lebanon and other middle-income countries in the community settings. Some of those studies have examined the cognitive function deterioration [13], the deficit accumulation [11], and the Study of Osteoporotic Fractures (SOF) Index [14]. Others evaluated the economic burden of caring for elderly population [15], mostly were conducted in high-income countries [11, 16] as well as in the institutions [13]. The aim of this study is to assess the psychometric properties of the Arabic version GFI in terms of feasibility, reliability, and construct validity in urban and rural populations in South Lebanon.

## Methods

### Study design and target population

From February to April 2015, a cross-sectional study was conducted among the elderly residing in urban and rural areas of Nabatieh in South Lebanon which enrolled 390 community-dwelling elderly individuals aged 65 years and above. Elderly, who had severe cognitive dysfunction or were very sick, were excluded from the study. A trained researcher identified eligible elderly persons and completed the questionnaire.

### Sample size

The sample size was determined by adopting the following statistical formula for minimum sample size calculation according to Yamane’s simplified formula [15, 17] that assumed *p* = 0.5, and 95% confidence interval:

$$ n=\frac{N}{1+N\ \left(e\times e\right)} $$

where *N* = the sampling frame (the total number of elderly people on the Nabatieh areas that was 16,249) [5], *e* = the margin of error (5% was used), and *n* = the minimum sample size of elderly persons. *p* = 0.5 (setting the response distribution to 50% is the most conservative assumption). The sample size needed equals 390.

### Sampling selection method

The sample was a probability-based, multi-stage cluster sample of 390 household elderly. They were set in 28 clusters. Sampling was at several levels: (i) city or village, (ii) neighborhood, (iii) household, and (iv) individual. A fixed sample size of exactly 40 households was selected from each cluster in urban areas, and 10–12 households in each cluster in rural areas. In the absence of reliable census data in the various communities, the random sampling of households was done according to the “itinerary method.” Overall, we selected 117 (30.0%) individuals in urban and 273 (70.0%) individuals in rural areas, taking into consideration the proportion of urban to rural residents in Nabatieh area. For each individual, we allocated a substitute individual, in case of refusal to participate.

### Data collection methods

A predesigned structured interview questionnaire was developed to collect the following data: sociodemographic data including demographic characteristics (age, gender, marital status, education, and residency location), living conditions (living alone or with others, having a housekeeper in the house), health insurance, and the financial perception (poor, fair, good, and very good).

Health status and lifestyle characteristics were assessed by self-rated health status (SRH), global quality of life (QoL) assessment, and declared morbidity. SRH and the perception of the QoL were showed to be reliable methods for measuring, respectively, the health status [18] and the quality of life concept [19]. SRH was assessed by one question, and the response was rated from 1 (very poor) to 6 (excellent); also, the overall QoL status was rated from 1 (very poor) to 5 (very good). Morbidities were recorded by asking participants (using a recall period of 12 months) if they suffered from chronic diseases (such as hypertension, diabetes, and insomnia). All self-reported morbidity data were checked for its accuracy from the responsible physician. In addition, the level of physical exercise was assessed by estimating the frequency and duration of exercise (excluding housework) by “a minimum of 30 min daily,” “occasionally,” or “never.” Information about the pastime activities and social club membership of the aged persons was also collected.

The Arabic version of Vulnerable Elders Survey (VES-13) is a tool used to assess the risk of health deterioration in community-dwelling older adults [20]. It consists of the following factors: age (0–3), self-reported health status (0–1), physical disability (0–2), and functional disability (0–4). It is a 13-item simple function-based questionnaire which is helpful for identifying vulnerable elderly persons in the community [20–23]. Each question on the test has a point scoring system beside it, and these numbers are totaled to give an overall score with the highest possible score of the scale is 10. A score greater than or equal to 3 classifies the participant as vulnerable. The VES-13 has been evaluated with good psychometric properties in Lebanon [24]. The Cronbach alpha coefficient for the overall scale of the VES-13 was 0.90, which is an indicator of the reliability of the scale. After excluding the age item, as is usually the case in some studies, the reliability coefficient was 0.91 [24].

The Groningen Frailty Indicator (GFI) [7, 12, 25] was developed in the Netherlands by Steverink and colleagues. It is a 15-item screening instrument for determining the level of frailty, which is available in a professional and self-report version. It measures the loss of functions and resources in 4 domains: physical (mobility functions, multiple health problems, physical fatigue, vision, and hearing), cognitive (cognitive dysfunction), social (emotional isolation), and psychological (depressed mood and feelings of anxiety). All answer categories were dichotomized, and a score of 1 indicated a problem or dependency. The GFI total score ranged from 0 to 15. A score of 4 or higher was considered as moderately to severely frail [25]. The GFI has been evaluated with good psychometric properties in Western Europe [12].

### Translation and cultural adaptation of the GFI

The translation and cultural adaptation of the GFI followed a standardized protocol [19]. A forward translation by two professionals (native Arabic speakers with excellent proficiency in English) was done. Apart from the translation, items were reviewed for cultural appropriateness and measurement equivalence. Because of the difficulties related to Arabic grammar and to the style of Arabic writing, two other Arabic linguistic experts also reviewed the translated version. Then, the Arabic version of the GFI was back translated by a native English speaker living in Lebanon, who was unaware of the original English language document. Once the backward translation was completed, the team reconvened to review and resolve the discrepancies between the backward translation and the original document. Finally, a pretest was conducted on a group (20 subjects) of lay native Arabic speakers. For each item, the group was asked to explain how it was understood. The participants indicated no difficulties in answering the items but preferred minor adaptation for one item of the social domain “Do you sometimes feel abandoned”: some persons expressed the desire that a distinction be made between family relationships, relations with neighbors, and social relationships (i.e., with friends) due to the importance of the family in oriental traditions. This pilot sample was excluded from the total sample.

### Statistical analysis

Mean and standard deviations were calculated for the total GFI scores and separate GFI domains since the data were normally distributed. Elderly persons were grouped according to a high (≥ 4) and low (< 4) GFI score. The differences between groups were tested with chi-square test, with a *p* value of < 0.05 to be considered significant.

The feasibility of the GFI was assessed by determining the proportion of missing values per item [12, 19, 25]. The reliability of the GFI and VES-13 scale scores was estimated using internal consistency methods (Cronbach’s alpha), whereas an alpha above 0.70 was acceptable for group comparisons [26–28]. The construct validity of the GFI was assessed in terms of known-groups validity and convergent and discriminant validity. For known-groups validity, we hypothesized that the GFI total scores differ among elderly subgroups as older people, women and those with comorbidities. To assess the construct validity of the GFI, we also calculated Spearman’s rank correlation coefficient between the GFI total score and concepts that measure similar concepts (convergent validity) and concepts that measure different concepts (discriminant validity). Therefore, we assumed positive correlations between the GFI and similar constructs (the VES-13) and negative correlations with total GFI scores versus different constructs, such as QoL and SRH [12].

Multivariate analysis was performed by stepwise logistic regression of the dichotomized GFI data (cutoff score < 4 versus ≥ 4) taking in consideration the gender, age, sport activity, social club membership, morbidity, QoL, and financial status perception to determine which characteristics were associated to the frailty among elderly. A variable is added to the model if the significance level is less than or equal 0.05.

All statistical analyses were performed by using SPSS version 22.0 (SPSS, Chicago, IL).

## Results

### Respondent characteristics

Out of the 400 households identified, 390 elderly persons were able to participate (97%). The duration of the total interview was 40–50 min. Concerning the GFI, it took 10–15 min to complete while VES-13 took 5 min. The sample included 199 (51%) women, and 273 (70%) lived in rural areas. The mean age was 76.11 (SD = 7.6) years. Nearly half of the sample (50.2%) was illiterate, and 39.3% were divorced or widowed. No one was living alone in their house. Most of the participants (81.8%) experienced development of morbidity (Table 1). The most predominant self-reported diseases were hypertension (50.0%), diabetes (37.9%), cardiovascular disease (31.3%), and joint and back pain (16.9%). Nearly half of the elderly reported their quality of life as fair (48.5%) and similarly their health status (45.6%).

**Table 1:** Sociodemographic and health characteristics of the community-dwelling elderly in South Lebanon in 2015 (*n* = 390)

Variables	Frequency (%)
Age, mean (SD)	76.1 (7.6)
Gender	
Women	199 (51.0)
Men	191 (49.0)
Residency	
Urban	117 (30.0)
Rural	273 (70.0)
Education level	
Illiterate	197 (50.5)
Elementary and lower	116 (29.8)
Intermediate and secondary	46 (11.8)
University and higher	31 (7.9)
Marital status	
Single	13 (3.3)
Married	224 (57.4)
Divorced, widowed	153 (39.3)
Current work	
Working	57 (14.6)
Not working	333 (85.4)
Financial perception	
Very poor and poor	56 (14.4)
Fair	189 (48.5)
Good	112 (28.6)
Very good and excellent	33 (8.5)
Quality of life	
Very poor and poor	34 (8.7)
Fair	189 (48.5)
Good and very good	167 (42.8)
Self-rated health status	
Very poor and poor	76 (19.5)
Fair	178 (45.6)
Good	113 (29.0)
Very good and excellent	23 (5.9)
Morbidity	
Yes	319 (81.8)
No	71 (18.2)

*SD* standard deviation

### Psychometric characteristics of the Groningen Frailty Indicator

No one refused to answer the questions of the GFI. There were no missing data or unintelligible items. Only half percent (0.5 %) of the elderly persons scored the maximum total score of 15, and about 1% (1.3%) scored the minimum GFI score. Cronbach’s alpha coefficient for the 15 GFI items was 0.81, and Cronbach’s alpha of the subscales was high for all subscales (range 0.77 to 0.81), except the social scale (0.56) (Table 2).

**Table 2:** Description and homogeneity of Groningen Frailty Indicator (*n* = 390)

Scale	No. of items	Mean (SD)	Minimum–maximum	Cronbach’s alpha
Total score	15	6.8 (3.4)	0–15	0.81
Physical	9	3.3 (2.4)	0–9	0.77
Cognitive	1	0.1 (0.3)	0–1	Not applicable
Social	3	2.0 (1.0)	0–3	0.56
Psychological	2	1.4 (0.8)	0–2	0.78

The VES-13 score of 3 or higher identified 62.8% of older persons at risk for health deterioration, and the mean score was 4.53. The correlation of the GFI with the VES-13 was very strong (*r* = 0.73) and statistically significant (*p* = 0.001). Inverse significant correlation was found between GFI total score and the QoL (*r* = − 0.22, *p* = 0.001), and also, inverse significant correlation was found between GFI total score and SRH (*r* = − 0.66, *p* = 0.001) (Table 3).

**Table 3:** Spearman’s correlation between the Groningen Frailty Indicator scale and related scales (*n* = 390)

	VES-13	QoL	SRH
GFI	0.73	− 0.22	− 0.66
*p* (2-tailed)	0.001	0.001	0.001

*VES-13* Vulnerable Elders Survey, *QoL* quality of life, *SRH* self-rated health status, *GFI* Groningen Frailty Indicator, *p p* value

The majority of elderly persons (81.3%) have a GFI score of 4 or more. We found that the frailty is more prevalent among women, illiterate, divorced, and widowed elderly. Increasing age, having a poor QoL, and reporting more than one morbidity were significantly associated with the rates of frailty in South Lebanon (*p* ≤ 0.001). There are no statistically significant differences in the mean frailty score according to the place of residence of the elderly persons (*p* = 0.07) (Table 4).

**Table 4:** Distribution of elderly subgroups according to their Groningen Frailty Indicator score (*n* = 390)

	GFI score (total)		GFI score (dichotomous)		
	Mean (SD)	*p*	Low (< 4), *N* (%)	High (≥ 4), *N* (%)	*p*
Total sample	6.77 (3.41)		73 (18.7)	317 (81.3)	
Age (years)		≤ 0.001			≤ 0.001
65–74	5.16 (2.98)		43 (30.5)	98 (69.5)	
75–84	7.23 (3.21)		26 (14.4)	155 (85.6)	
≥ 85	8.90 (3.27)		4 (5.9)	64 (94.1)	
Gender		≤ 0.001			≤ 0.001
Female	7.67 (3.28)		22 (11.1)	177 (88.9)	
Male	5.83 (3.30)		51 (26.7)	140 (73.3)	
Residency location		0.07			0.25
Urban	6.29 (3.31)		26 (22.2)	91 (77.8)	
Rural	6.97 (3.44)		47 (17.2)	226 (82.8)	
Education		≤ 0.001			≤ 0.001
Illiterate	7.66 (3.21)		21 (10.7)	176 (89.3)	
Elementary and lower	6.53 (3.22)		24 (20.7)	92 (79.3)	
Intermediate and secondary	5.50 (3.53)		14 (30.4)	32 (69.6)	
University and highest	3.84 (2.86)		14 (45.2)	17 (54.8)	
Marital status		≤ 0.001			≤ 0.001
Single	7.00 (3.63)		3 (23.1)	10 (76.9)	
Married	5.87 (3.32)		59 (26.3)	165 (73.7)	
Divorced, widowed	8.07 (3.10)		11 (7.2)	142 (92.8)	
Health coverage		0.001			0.37
Yes	6.37 (3.24)		53 (19.9)	213 (80.1)	
No	7.62 (3.63)		20 (16.1)	104 (83.9)	
Self-rated health status		≤ 0.001			≤ 0.001
Very poor and poor	10.32 (2.57)		1 (1.3)	75 (98.7)	
Fair	7.28 (2.59)		13 (7.3)	165 (92.7)	
Good	4.34 (2.60)		45 (39.8)	68 (60.2)	
Very good and excellent	3.09 (2.17)		14 (60.9)	9 (39.1)	
Financial perception		≤ 0.001			≤ .0001
Very poor and poor	8.93 (3.10)		1 (1.8)	55 (98.2)	
Fair	7.17 (3.20)		24 (12.7)	165 (87.3)	
Good	5.27 (3.18)		38 (33.9)	74 (66.1)	
Very good and excellent	5.91 (3.47)		10 (30.3)	23 (69.7)	
Quality of life		≤ 0.001			≤ 0.001
Very poor and poor	8.32 (3.33)		3 (8.8)	31 (91.2)	
Fair	7.19 (3.22)		24 (12.7)	165 (87.3)	
Good and very good	5.98 (3.47)		46 (27.5)	121 (72.5)	
Practicing sportive activity		≤ 0.001			≤ 0.001
Yes, regularly	3.88 (2.65)		28 (47.5)	31 (52.5)	
Yes, irregularly	5.10 (2.91)		20 (38.5)	32 (61.5)	
No	7.69 (3.18)		25 (9.0)	254 (91.0)	
Social club membership		0.001			0.01
Yes	4.91(3.60)		12 (35.3)	22 (64.7)	
No	6.95 (3.34)		61 (17.1)	295 (82.9)	
Morbidity		≤ 0.001			≤ 0.001
Yes	7.33 (3.22)		41 (12.9)	278 (87.1)	
No	4.24 (3.11)		32 (45.1)	39 (54.9)	
Number of Morbidities		≤ 0.001			≤ 0.001
0	4.24 (3.11)		32 (45.1)	39 (54.9)	
1–3	7.27 (3.34)		38 (14.1)	231 (85.9)	
4 and plus	7.66 (2.50)		3 (6.0)	47 (94.0)	
Having housekeeper in the house		0.30			0.98
Yes	7.11 (3.83)		16 (18.8)	69 (81.2)	
No	6.68 (3.29)		57 (18.7)	248 (81.3)	

*GFI* Groningen Frailty Indicator, *SD* standard deviation, *N* frequency

Multivariate regression analysis showed that the degree of frailty in females is higher than in males (OR = 1.92; 95%CI = 1.01–3.71, *p* = 0.05). Frailty was more frequent in older people (*p* < 0.001) and far less frequent in subjects practicing sportive activities in their leisure time compared to those who were not physically active (OR = 3.10, 95%CI = 1.49–6.45, *p* = 0.002). Frailty was associated with morbidities and was more frequent in subjects who reported a poor financial status (OR = 20.06, 95%CI = 2.06–213.75, *p* = 0.01) (Table 5).

**Table 5:** Relationship between the Groningen Frailty Indicator scores and the elderly subgroup characteristics (*n* = 390)

	Exp (β)	*p* value	95% CI for Exp (β)	
			Lower	Upper
Constant	0.01	0.01		
Age	1.07	0.005	1.02	1.11
Gender		0.05		
Female	1.92		1.01	3.71
Male	1			
Quality of life		0.71		
Very poor and poor	0.60	0.51	0.14	2.66
Fair	1.10	0.80	0.54	2.23
Good and very good	1			
Practicing sportive activity		0.002		
No	3.10		1.49	6.45
Yes, regularly	1			
Financial perception		0.003		
Poor	20.98	0.01	2.06	213.75
Fair	2.69	0.06	0.95	7.63
Good	0.87	0.76	0.33	2.24
Very good and excellent	1			
Morbidity		0.001		
No	0.23		0.12	0.44
Yes	1			
Social club membership		0.53		
Yes	0.70		0.24	2.08
No	1			

Nagelkerke R square = 0.361; *CI* confidence interval. Variables shown in this table were selected because of their association with GFI in the univariate analysis and of their potential public health interest regarding the current situation in Lebanon. Marital status and education were excluded in view of their high association with gender (*p* < 0.001). SRH was also excluded in view of the high correlation with financial status (*r* = 0.53; *p* < 0.001)

## Discussion

This study has undertaken rigorous processes to translate and culturally adapt the GFI to the Lebanese Arab context; the Arabic version of the scale was evaluated and showed good psychometric properties.

Concerning feasibility, it was in general very good, no disturbing questions, no confusing items, and no missing data for items and scales, and the duration of administration of the questionnaire was short (10–15 min). This reinforces the expected validity (face validity) and therefore makes it possible to confirm the absence of problems related to translation [19].

The internal consistency generally exceeded the recommended minimum alpha coefficient standard for group comparison of 0.70 [26, 29] and also was higher than the reliability of the GFI in Dutch (0.68) [12] and Romanian study (0.746) [30]. Indeed, Ware [29] considered the reliability of 0.5 or above was acceptable. The presence of a relation between the dimensions of GFI and the sociodemographic and clinical characteristics is an important finding as such instruments could be used in therapeutic evaluations [12, 19] and supported the construct validity of the instrument. Construct validity was also evaluated by relating the GFI scale with the VES-13, QoL, and SRH. Our results were consistent with previous work showing that correlations of the GFI and other instruments showed evidence for both its convergent and its discriminant validity [7, 12].

The mean frailty score (6.77) using the GFI among the elderly in the study was higher than their Dutch [7, 12] and Romanian [30] counterparts. Drubbel et al. [7] and Peters et al. [12] found a mean score of 3.2 (*n* = 623; mean age 73.4 years) and 4.5 (*n* = 353, mean age = 81 years), respectively, in community-dwelling older patients in the Netherlands. Olaroiu et al. [30] reported a mean frailty score of 5.5 (*n* = 215; mean age 75 years) among independent old persons living in rural and urban areas in Romania.

The prevalence of frailty in South Lebanon (81.3%) was higher than middle- and low-income countries where the estimated prevalence of frailty was the highest in India (55.5%) and Ghana (40.8%), and the lowest in China (13.1%) [31]. The estimated prevalence of frailty in our study is different from those observed in a cross-sectional study performed in 10 European countries that revealed that 17% of the individuals aged at least 65 years were frail [32]. Also, the results obtained in our study are much higher than those of a study of a sample of older persons aged 70 years or older in the Netherlands where the percentage of frailty was between 46.3% on the GFI scale and 60% on the Sherbrooke Postal Questionnaire [33]. Another previous study found 39% of patients had a GFI score of 4 or higher in the Netherlands [7]. Compared with the old Romanians, the prevalence of frailty in independently living old Romanians was slightly lower where 75% had a GFI score of 4 or above [30]. Indeed, the 40–60% rate is a high prevalence of frailty in an elderly community [33], and it is likely to be an indicator of substantial costs associated with caregiving for the elderly [15]. Differences among frailty prevalence estimates between countries are probably due to a number of factors; for instance, lifestyles, health statuses, and demographic and socioeconomic characteristics vary greatly between countries at different stages of development. Prevalence of frailty is challenging [34] and indicates an increased need for integrated care [7].

The study findings supported health inequalities according to gender and age. In order to explain the emergence and persistence of health inequalities, social scientists rely primarily on two theories. The root cause theory explains how people in higher social classes have more ways to avoid health risks and pay for treatment, thereby contributing to better health. The second theory is the theory of cumulative advantages which note that the relative advantages become cumulative benefits throughout life, and ultimately producing inequities [35].

The present work revealed that age, gender, education, perceived financial status, physical inactivity, and comorbidities were associated with frailty. The results are indicative of the discriminative ability of the GFI. Our results were consistent with those of the SHARE study conducted in European countries [32] where women were more likely to be frail than men and increasing age positively influenced the probability of frailty, while additional years of education had a negative effect on this probability. Frailty increases with age because as people age, they accumulate deficits [34]: aging process generates limitations for carrying out some activities [15], and elderly become more vulnerable to adverse health outcome challenges [34]. A greater prevalence of frailty is expected in the female population because women live longer and generally have a larger number of comorbidities [32]. Also, the poor financial perception is more prevalent in frail elderly persons as poverty or precariousness is a major risk factor for poor health outcomes that are usually more prevalent in rural settings [36].

### Limitations of study

There were some potential limitations of the current study. It was carried out in only one area of Lebanon because of a lack of resources which limits the results to a certain region. The design effect of a cluster sample was not calculated. Also, this is a cross-sectional study which cannot establish a temporal relationship between individual characteristics and frailty.

## Conclusion

The GFI Arabic version has been evaluated as a valid and reliable instrument in rural and urban Lebanese elderly populations.

## Data Availability

Data will be available on reasonable request. Please contact the corresponding author for data requests.

## Abbreviations

CI: Confidence interval
Exp: Exponential
GFI: Groningen Frailty Indicator
OR: Odds ratio
*p*: *p* value
QoL: Quality of life
SD: Standard deviation
SRH: Self-rated health status
UNDP: United Nations Development Program
VES-13: Vulnerable Elders Survey
WHO: World Health Organization
